# Histone succinylation and its function on the nucleosome

**DOI:** 10.1111/jcmm.16676

**Published:** 2021-06-23

**Authors:** Jiayi Liu, Yu Shangguan, Donge Tang, Yong Dai

**Affiliations:** ^1^ Clinical Medical Research Center Guangdong Provincial Engineering Research Center of Autoimmune Disease Precision Medicine Shenzhen Engineering Research Center of Autoimmune Disease The First Affiliated Hospital(Shenzhen People’s Hospital), Southern University of Science and Technology Shenzhen China; ^2^ School of Medicine Southern University of Science and Technology Shenzhen China; ^3^ Guangxi Key Laboratory of Metabolic Disease Research Central Laboratory of Guilin, 924st Hospital Guilin China

**Keywords:** histone, succinylation, desuccinylation, nucleosome

## Abstract

Protein post‐translational modifications (PTMs) of histones are ubiquitous regulatory mechanisms involved in many biological processes, including replication, transcription, DNA damage repair and ontogenesis. Recently, many short‐chain acylation histone modifications have been identified by mass spectrometry (MS). Lysine succinylation (Ksuc or Ksucc) is a newly identified histone PTM that changes the chemical environment of histones and is similar to other acylation modifications; lysine succinylation appears to accumulate at transcriptional start sites and to correlate with gene expression. Although numerous studies are ongoing, there is a lack of reviews on the Ksuc of histones. Here, we review lysine succinylation sites on histones, including the chemical characteristics and the mechanism by which lysine succinylation influences nucleosomal structure, chromatin dynamics and several diseases and then discuss lysine succinylation regulation to identify theoretical and experimental proof of Ksuc on histones and in diseases to inspire further research into histone lysine succinylation as a target of disease treatment in the future.

## 1 INTRODUCTION

Histone modifications are dynamic and reversible, and they regulate chromatin structure, the DNA unwrapping rate and protein binding to histones, playing vital roles in regulating all chromatin‐based processes, including gene expression, transcription and DNA damage repair.^1^, ^2^ More than 16 kinds of histone modifications have been identified, such as methylation, acetylation, phosphorylation, ubiquitination and succinylation.^2^, ^3^, ^4^ Lysine succinylation is a newly discovered post‐translational modification on histones.^5^, ^6^, ^7^ This review is based on a survey of the research on the succinylation on histones and nucleosomes in recent years and discusses the specific role of succinylation in gene transcription, DNA repair, disease occurrence and disease development.

## 2 IDENTIFICATION AND DISTRIBUTION OF LYSINE SUCCINYLATION ON HISTONES

### 2.1 Identification of lysine succinylation on histones

Lysine succinylation (Ksuc) was first observed at the active site of homoserine transsuccinylase, which was considered an intermediate reaction of succinyl group transfer from succinyl‐CoA to homoserine.^8^ The N^ε^‐ (succinyl) lysine (SUL) adduct is a product of the peroxidation of docosahexaenoic acid (DHA) in vitro and in vivo, and SUL bovine serum albumin (BSA) is present during the oxidation of DHA.^9^ These studies identify the key compound in succinylation. However, it was unclear whether the succinyl group or methylmalonyl group caused the mass change because of the lack of supporting evidence to distinguish succinyl groups and their isomers. Upon the established use of western blot analysis,^10^ labelling of isotopic succinate in vivo, tandem MS (MS/MS) and HPLC coelution to identify synthetic counterparts and obtain lysine succinylation, the decisive supporting evidence was found^10^: Zhang et al identified and characterized 14 kinds of *E coli* proteins and 69 succinylated lysine sites. This study provided decisive evidence showing that lysine succinylation is a natural and novel post‐translational modification. Subsequently, scientists found histone lysine succinylation in mammalian proteins,^7^ HeLa,^5^ mouse embryonic fibroblasts^5^ and histone lysine succinylation was also found in Drosophila S2 cells and *Saccharomyces cerevisiae* cells,^5^ demonstrating that histone succinylation is a highly evolved conserved mark in eukaryotes. The discovery of lysine succinylation also contributed to the discovery of malonylation (Kma)^5^ and glutarylation (Kglu).^11^

### 2.2 Distribution of lysine succinylation on histones

**FIGURE 1:**
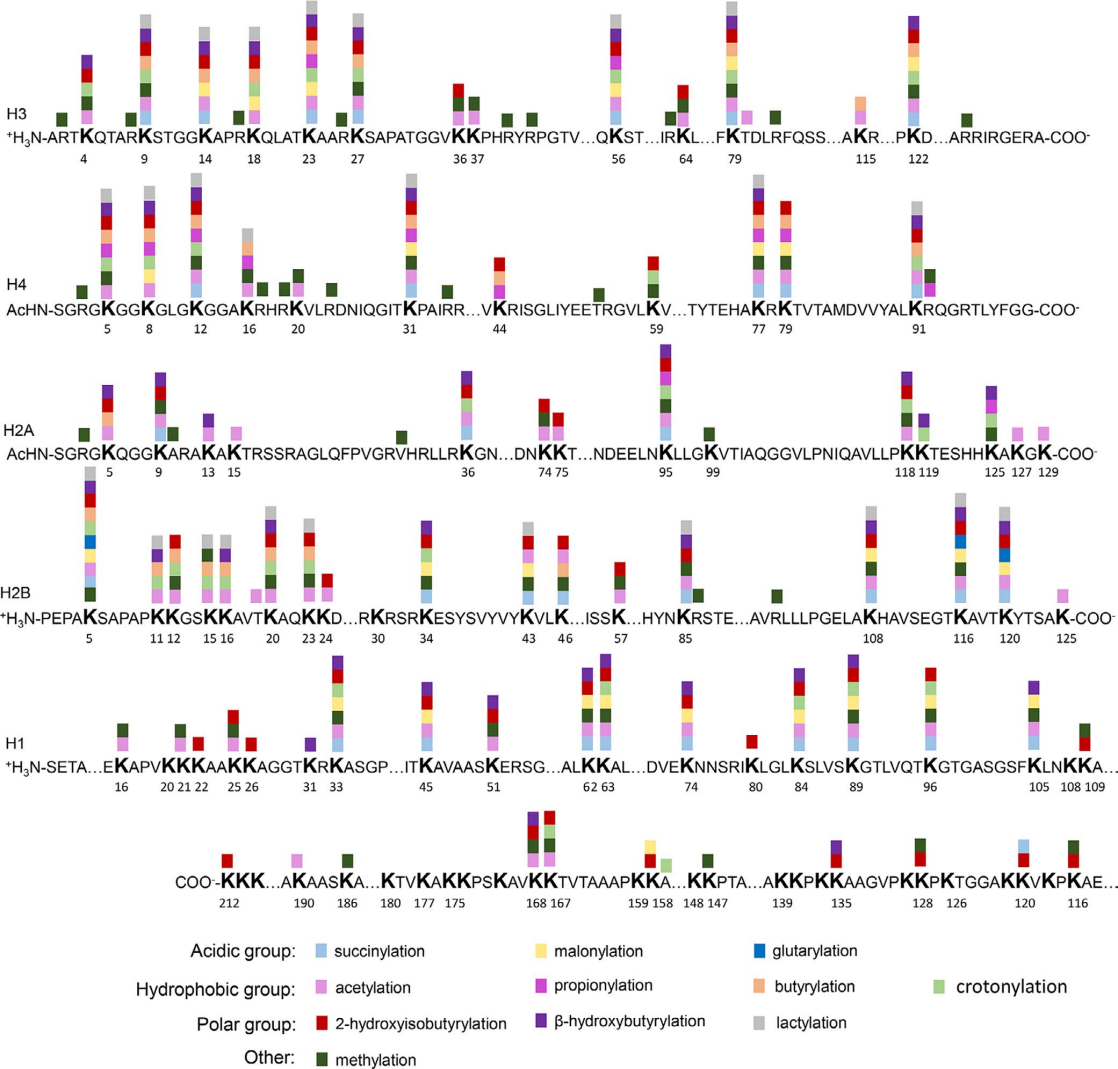
Distributions of acylation and methylation on histones. This figure mainly focuses on identified lysine succinylation sites on histones, and not all other histone modifications are shown (Reference 3, 16, 17, 63, 68, 69)

Lysine succinylation is mostly localized in mitochondria, followed by the cytoplasm and nucleus. Succinyl‐CoA and lysine succinylation are abundant in tissues with numerous mitochondria, including the heart, brown adipose tissue and liver.^6^, ^12^, ^13^, ^14^, ^15^ A moderate bias toward succinylation was found in helical regions, and a moderate bias away from succinylation was found in coiled regions in four organisms.^6^ Studies on histones have revealed lysine succinylation sites in both linker histone H1 and four core histones H2A, H2B, H3 and H4. Moreover, there are conserved sites in several species. Most of the succinylation sites identified in vivo are in the globular domain and C‐terminal of histones not the N‐terminal tail. These sites seem to be in regions that largely overlap with other sites of PTM, such as acetylation, methylation and 2‐hydroxyisobutyrylation (Figure 1).^5^, ^16^, ^17^

## 3 THE STRUCTURE AND FUNCTION OF HISTONE KSUC IN THE NUCLEOSOME

### 3.1 Changes in the structure of the nucleosomes induced by histone Ksuc

Lysine succinylation refers to the process of covalently binding a succinyl group (‐ CO‐CH2‐CH2‐COOH) to the ε‐amine of lysine with the enzymatic or non‐enzymatic mechanisms.^10^, ^16^ Lysine succinylation can result in a changed charge state of lysine residues from +1 to −1 at physiological pH, comparable to the change in charge state, from 0 to 2 charge, induced by protein phosphorylation of serine, threonine or tyrosine residues.^10^, ^16^ Simultaneously, succinylation on lysine may induce more significant structural changes in space than methylation or acetylation modifications.^5^, ^16^

PTMs on histones can directly alter chromatin packaging by changing the chemical structure of the histone or internuclear interactions caused by the change in the net charge, hydrogen bond, molecular size or substrate PTM residue hydrophobicity. Each core histone field contains a spherical core structure and a series of highly conserved lysine residues. The tail end of the flexible ε‐amine histone lysine residue baseband has a positive charge that can form a salt bridge with the negatively charged DNA skeleton. Acetylation is a more in‐depth acylation, and research shows acetylation on histone H4K91 residues at the interface of the (H3‐H4)_2_ tetramer and H2A‐H2B dimer hinders the histone interactions, undermining the stability of the nucleosome.^18^ Moreover, H3K56 acetylation sites enhance the hydrophobicity, and increase the size of the lysine side chain, reduce the tail affinity for chromatin, and expose a larger sequence of DNA.^2^, ^19^, ^20^, ^21^ Based on the similarity of charge changes in Ksuc (from +1 to −1) and Kac (from +1 to 0), Ksuc produces a negative charge, Kac produces a zero charge, and Ksuc produces a moiety with a larger volume,^22^ but the effect of Ksuc on histones may be similar to that of Kac. On the one hand, a histone mutation in budding yeast mimics Ksuc^23^ by mutating lysine to glutamate, and the mutation of two succinylated sites, H3K79 and H4K77, reduces the stability of nucleosomes on chromatin structure and inhibits both telomere silencing and rDNA silencing in vivo.^5^ Moreover, histone H4 was synthesized by an expressed protein ligation (EPL) strategy and site‐specific succinylation of H4K77^24^ based on the chemical method developed in Jing et al for early application of the site‐specific incorporation of H2BK34 into a succinylation mimic.^25^ H4K77suc led to the formation of a larger proportion of (H3‐H4)_2_ tetramers and DNA complexes, while unmodified H2B and H2A were mainly assembled into an intact mononucleosome. These results show that H4K77suc can interfere with nucleosome assembly. Further experiments revealed that H4K77suc weakened the DNA interaction with the H2A‐H2B dimer, leading to a significant decrease in nucleosome stability in vitro, increasing the DNA unwrapping rate on the surface of histones and promoting transcription. In addition, the outcome of mutating H4K77 to glutamate in vivo was consistent with that of a previous phenotypic study of the H4K77 mutant in budding yeast.^5^, ^26^ Previously, the site‐directed succinylation of H2BK34 in vitro also showed that Ksuc destroys the stability of the nucleosome by affecting the interaction of DNA and histone, which is consistent with the structural defects of telomeres and global chromatin in yeast H2BK37E mutant cells.^25^ Moreover, to research the genomic localization of the succinyl‐lysine modification in chromatin, it is found that lysine succinylation was enriched at the gene promoter by ChIP‐seq. The bimodal pattern of peak enrichment flanking the transcriptional initiation site was identified in the whole genome, suggesting a role for Ksuc in transcriptional regulation. It also verified a strong correlation between gene expression and protein succinylation near the transcriptional start site by showing a correlation between the succinyl‐lysine ChIP signal and gene expression, which further indicates that promoter succinylation is a mark of gene transcriptional activity.^27^ Recently, it was also found that H3K122succ is enriched at transcription start sites and can be a mark of active genes, stimulating transcription in vitro and changing the structure of the stable nucleosome.^28^

These experiments all prove that histone Ksuc is closely related to gene expression in vitro, similar to Kac, by weakening the affinity between DNA and histones to reduce the stability of the nucleosome and chromosomes and to promote the separation of DNA and protein, which can promote the binding of transcription factors to DNA and then contribute to transcription. However, there are still many questions regarding how succinylation regulates the stability, repair and kinetics of the nucleosome and chromosomes; for example, the genes promoted by histone succinylation in vivo still need to be identified using experimental evidence, and whether the Ksuc of histones in vivo is indeed an essential mark of DNA repair needs further elucidation.

### 3.2 The functional role of histone Ksuc in the nucleosome

#### 3.2.1 The source of histone succinylation in the nucleosome

Current studies have verified a quantitative correlation between metabolic processes and different histone acylation modifications, and the total concentration of metabolites is consistent with the relative abundance of lysine acylation.^6^, ^29^ Most groups added to histones during acylation are produced from acyl‐CoA, and the donor of acyl‐CoA determines the level of histone acylation. A series of studies with eukaryotic cells have suggested that histone modification is sensitive to changes in the intracellular concentration of metabolic intermediates, linking cellular metabolism to epigenetic changes.^30^, ^31^

Studies have suggested and reviewed that the moiety added to histones upon succinylation is produced from succinyl‐CoA, while intracellular succinyl‐CoA is mainly synthesized in mitochondria and is produced from α‐ketoglutarate, succinyl‐carnitine and succinate.^32^ Succinate has also been observed in peroxisomes in some studies.^32^

Weinert et al suggested that in budding yeast (*S cerevisiae*), the induction of galactose contributes to the level of succinyl‐CoA, thus significantly increasing the global succinylation level, while a lack of Lsc1 inhibits succinyl‐CoA conversion to succinate, thus significantly decreasing global succinylation levels, which provides direct evidence that the concentration of succinyl‐CoA produced from tricarboxylic acid(TCA) affects the level of succinylation in various compartments of the whole cell.^6^ Succinyl‐CoA produced in mitochondria is the succinyl donor to lysine, and it may pass through the mitochondrial membrane or be produced in the cytoplasm and nucleus during synthesis with succinyl coenzyme A.^6^ Acyl‐CoA metabolites can be transferred from the cytoplasm to the nucleus through small‐molecule‐permeable nuclear pores,^33^ as corroborated by experiments showing that the addition of acyl‐CoA to the isolated nucleus affects the corresponding chromatin acylation.^29^, ^34^ The oxoglutarate carrier SLC25A11 transports α‐ketoglutarate from mitochondria.^35^, ^36^ Upon nuclear localization, the α‐KGDH complex catalyses the conversion of α‐ketoglutarate to succinyl coenzyme A in the nucleus, and then KAT2A (GCN5) can exert succinyltransferase activity and succinylate a histone lysine.^37^

Another mechanism of succinyl group output from mitochondria involves carnitine shuttling. Higher succinyl‐carnitine levels in patients’ blood with SUCL (succinyl‐CoA ligase) subunit gene mutations constitute another piece of supportive evidence.^38^ However, succinyl‐CoA is neither the substrate of carnitine acetyltransferase (CrAT) nor the succinyl‐carnitine transformed product.^39^ Carnitine palmitoyltransferase 1A (CPT1A) is a lysine succinyltransferase (LSTase) in vivo and in vitro. It can regulate the enzyme activity and metabolism of substrate proteins independent of its classical carnitine palmitoyltransferase (CPTase) activity. Moreover, in the CPT1AG710E mutant, CPTase activity was inactivated, but not LSTase activity.^40^ Therefore, the succinyl‐carnitine shuttle from mitochondria may not follow the classical pathway, and the transformation of succinyl‐carnitine to succinyl‐CoA in the nucleus‐cytoplasmic chamber remains to be studied.

In addition, the level of succinate increases the level of succinylation of intracellular proteins. Treatment of mouse embryonic fibroblasts with 3‐nitropropionic acid (3‐NPA),^13^ an inhibitor of succinate dehydrogenase (SDH), slightly elevated the accumulation of protein succinylation, suggesting that decreased SDH may result in increased levels of succinate and protein succinylation. Additionally, Li et al revealed that succinylation is related to an increase in succinate.^41^ Succinate is synthesized and transported from mitochondria to the cytoplasm in some ways. Succinate can be exported from mitochondria through the dicarboxylate carrier SLC25A10^42^, ^43^ and synthesized in the cytoplasm as a by‐product of α‐ketoglutarate‐dependent enzymes.^44^ Succinate in the nuclear and cytoplasmic compartments originates in peroxisomes. The succinate increases in the content of peroxisomal thioesterase ACOT4, which is highly expressed in the kidney, liver and intestine. Compared to other acyl‐CoA substrates, ACOT4 tends to hydrolyse succinyl‐CoA to succinate.^45^, ^46^ Non‐selective peroxisomal membrane channels, such as PXMP2, can transmit the small succinate molecule but not large succinyl‐CoA molecule.^47^ Nevertheless, in the nucleus and cytoplasm, the function of succinate from multiple sources remains to be determined on the protein succinylation.

Genetic and pharmacological targeting of candidate enzymes and transporters combined with isotope tracing is necessary to address the origin and regulation of nuclear and cytoplasmic succinyl‐CoA.^32^ Mitochondria have the most abundant succinyl groups. Whether mitochondria can change membrane permeability to release succinyl materials and succinyl‐CoA producing enzymes to cytoplasm and nucleus remains to be further studied. The role of excessive protein succinylation in disease is also an issue to be researched. Succinyl‐CoA is produced locally in the nucleus. Moreover, when the nuclear membrane disappears during mitosis, metabolites and metabolic enzymes can approach histones, and whether the succinylation of histones is correlated with cell division. For most histone acylation marks, including histone lysine succinylation, the mechanism of acyl‐CoA production in the nucleus‐cytoplasmic compartment remains poorly understood.

#### 3.2.2 Succinylation of lysine in histones

Compared with the enzyme‐mediated succinylation and desuccinylation of proteins in mitochondria, lysine succinylation of histones in the nucleus is less characterized. However, experimental evidence suggests that enzymes and non‐enzymes can mediate Ksuc of histones. However, there is controversy about histone Ksuc mediated by enzymes.

Many studies suggest that enzymes can catalyse histone succinylation. Research shows that p300 succinylates a synthetic peptide in the histone tail sequence in vitro.^11^, ^22^, ^28^ Atsushi Yokoyama et al offered sophisticated evidence showing that histone succinylation in the nucleus is an enzymatic process. Although this study extracted histones from HepG2 cell nuclei as the histone succinylation substrates, which did not show Ksuc activity, it was found that enzyme‐mediated histone succinylation indeed occurred in an SP 1.0 M KCl fraction of nuclear extracts.^48^ In addition, Wang et al showed that the affinity of N‐acetyltransferase 2A (KAT2A, also known as GCN5) for succinyl‐CoA is higher than for acetyl‐CoA. The α‐ketoglutarate dehydrogenase (α‐KGDH) complex consists of three parts: oxoglutarate dehydrogenase (OGDH; E1), dihydrolipoyl succinyltransferase (DLST; E2) and dihydrolipoyl dehydrogenase (DLD; E3). Α‐KGDH in the nucleus produces succinyl‐CoA, which provides materials for KAT2A to mediate histone succinylation. The mutation of tyrosine 645 in KAT2A ring 3 reduced H3 succinylation without changing H3 acetylation.^37^ A recent study revealed that histone acetyltransferase 1 (HAT1) is a novel histone and non‐histone succinyltransferase but does not participate with transferases of other PTMs, such as butyrylation and crotonylation, in HepG2 cells.^49^ The study also suggested that HAT1 may be more likely to combine with succinyl‐CoA than with acetyl‐CoA.^49^

In contrast, other findings show that enzymes may not play a central role in histone succinylation. Simithy et al studied succinylation on histone H3 and H4 by seven kinds of histone acetyltransferases (HATs) and non‐enzyme conditions in vitro.^29^ To determine substrate specificity, H3 was assayed with HATs p300, CBP, PCAF and KAT2A, and histone H4 was assayed with HATs MOF, NatA and Tip60.^29^ It was found that non‐enzymatic action mainly mediated histone lysine succinylation, and increasing the succinyl‐CoA concentration increased the abundance of succinylation in the nucleus. Acid acyl‐PTMs, including malonylation, succinylation and glutarylation, are most prone to non‐enzymatic catalysis in the absence of enzymes, and the non‐enzymatic acylation sites are commonly close to the C‐terminal of histones, that is, H3K56‐K122 residues and H4K59‐K91 residues, which is consistent with the site of lysine succinylation on histones. However, at the same concentration, HATs preferentially function with acetyl‐CoA rather than with other acyl coenzymes such as succinyl‐CoA. Enzymes utilize other cofactors, mainly depending on the size of acyl groups confirming the recent studies that the active sites in p300 and KAT2A are structurally incompatible with long‐chain acyl donors.^50^, ^51^ Lara Zorro Shahidian et al also suggested that p300/ CBP can succinylate H3K122 preferentially with acetyl‐CoA.^28^

The different results of enzyme‐mediated succinylation, such as KAT2A, may be due to site specificity and experimental methods (qualitative and quantitative). Wang et al focused on H3K79 succinylation mediated by KAT2A by using succinyl‐lysine and anti‐H3K79Suc antibodies in western blot analysis and performed mass spectrometry qualitative identification.^32^, ^37^ Simithy et al reported the average acylation of 14 lysine sites on histone 3, including H3K79, and detected histone acylation by quantitative mass spectrometry.^29^, ^32^

Under various proofs of enzymatic and non‐enzymatic functions, it may be possible that most succinylation is realized non‐enzymatically and that enzyme‐catalysed succinylation is carried out under special biochemical conditions and at specific sites in vivo. Moreover, there is still a large gap in the reads associated with Ksuc on histones, and it has only been reported that glioma‐amplified sequence‐41 (GAS41) binds histone succinylation sites in a pH‐dependent manner, and GAS41 may be a lysine succinylation reader in yeast.^52^

##### KAT2A‐mediated histone succinylation, gene expression and diseases

Though the studies of KAT2A‐mediated histone succinylation at lysine residues in the nucleus are at an early stage, specific data demonstrate that it may be a crucial part in the development of diseases such as tumours, human pancreatic ductal adenocarcinoma (PDAC) and hepatitis B virus.

KAT2A plays a vital role in succinylating histones and regulating gene expression. KAT2A is a histone acetyltransferase (HAT)^53^, ^54^ and catalyses histone succinylation.^37^ Lysine79 on histone H3, a high‐frequency amino acid around transcription start sites (TSSs), has been found to be succinylated by KAT2A.

With respect to the mechanism of KAT2A‐mediated succinylation on histones, studies indicate that whatever prohibiting the α‐KGDH complex from entering the nucleus, or blocking the α‐KGDH complex from entering the nucleus, or expressing mutant KAT2AY645A reduced histone succinylation on H3K79 and suppressed the growth of intracranially injected glioblastoma cells, indicating that KAT2A‐mediated H3K79 succinylation promotes tumour growth. These results reveal a fundamental histone modification mechanism and illustrate that the local generation of succinyl‐CoA by the nuclear α‐KGDH complex coupled with the succinyltransferase activity of KAT2A promotes histone succinylation, which induces gene expression changes, tumour cell proliferation and tumour growth.^37^

Furthermore, the relationship between high KAT2A expression in PDAC specimens and advanced stages of PDAC in patients is positive and associated with poor survival. In PDAC specimens, H3K79 succinylation by KAT2A in the promoter region of YWHAZ (encoding 14‐3‐3ζ) benefits YWHAZ and 14‐3‐3ζ expression, thereby restricting the degradation of β‐catenin.^55^ In the presence of 14‐3‐3ζ, β‐catenin shows diminished ubiquitylation and degradation, increased stability and disassociation from the binding with β‐TrCP E3 ligase. Moreover, 14‐3‐3ζ upregulates not only β‐catenin expression but also downstream gene expression in the cytoplasm and nucleus.^56^, ^57^ The depletion of KAT2A succinyltransferase activity by the KAT2A Y645A mutant reduces H3K79 succinylation and 14‐3‐3ζ expression, leading to harm β‐catenin stability and subsequently decreasing expression of cyclin D1, c‐Myc, GLUT1 and LDHA.^55^ KAT2A‐mediated H3K79 succinylation promotes 14‐3‐3ζ and β‐catenin expression, which contributes to glycolysis, cell proliferation, migration and invasion of PDAC cells upon epithelial‐to‐mesenchymal transition.^55^ These findings reveal a novel and instrumental role of KAT2A‐mediated histone succinylation in regulating gene expression and disease.^55^

Recently, succinylation of both histone H3K79 and hepatitis B virus covalently closed circular DNA (HBV cccDNA) by KAT2A has been identified in HBV‐infected human liver‐chimeric mice and HBV‐expressing cell lines. KAT2A knockdown can limit KAT2A binding to HBV cccDNA and reduce the levels of HBV DNA, HbsAg and HbeAg in the supernatant of HepaRG cells infected with de novo HBV. Furthermore, the inhibition of KAT2A succinylation reduces H3K79 succinylation, thereby restricting HBV replication. These phenomena were verified in the reduction of cccDNA expression in cells expressing siKAT2A.^58^ In addition, KAT2A‐mediated succinylation of histone H3K79 was found to contribute to epigenetic regulation of cccDNA microchromosomes. Experimental data showed that IFN‐α affects the epigenetic regulation of HBV cccDNA minichromosomes in de novo HepG2‐NTCP and HBV‐infected HepG cells by inhibiting the succinylation of KAT2A‐mediated histone H3K79 to clear HBV cccDNA. These findings provide a new horizon for studying the mechanism by which KAT2A‐mediated succinylation regulates the minichromosome epigenetic regulation of HBV cccDNA and revealing the mechanism by which IFN‐α inhibits cccDNA.^58^

Although studies about the relationship of KAT2A‐mediated histone succinylation and diseases need further clarification, KAT2A‐mediated histone acylation has been reported to be correlated with the tumour development processes. For example, long non‐coding RNA (lncRNA) GClnc11 can serve as a protein scaffold for recruiting WDR5 and the KAT2A complex to the SOD2 promoter, which can elevate the histone modifications level H3K4 trimethylation and H3K9 acetylation near the promoter.^59^ These studies verify that KAT2A‐succinyl‐CoA structural conformations are different than KAT2A‐acetyl‐CoA conformations.^37^, ^55^, ^59^ The abovementioned findings suggest that histone succinylation is similar to other PTMs and acts as a significant epigenetic mark but is different from them in the mechanism of regulation, which also provides new evidence and ideas for the study of histone succinylation as a new therapeutic target.

##### The effect of hypersuccinylation caused by SDH deletion in cancer

Alterations in succinate dehydrogenase (SDH) expression and the function of its encoded product have been found in numerous tumours. Succinate is a competitor of α‐ketoglutarate and a competitive inhibitor of multiple α‐ketoglutarate‐dependent dioxygenases, such as histone demethylases and prolyl hydroxylases. Previous studies have revealed that mutations in SDH tumour suppressors^60^ or SDH loss^27^ increase succinate and protein succinylation. Knockdown of the SDH gene enhances the level of succinate, resulting in reduced activities of α‐ketoglutarate‐dependent dioxygenases. Ectopic expression of the SDH mutant in tumour samples inhibits histone demethylation and hydroxylation of 5mC. These changes suggest that defective SDH contributes to gene‐wide and histone methylation, which may positively enhance tumour growth.^60^ Moreover, histone succinylation at H3K79 contributes to transcription and tumour development.^37^ However, another recent study suggested that increasing protein succinylation by dimethyl succinate (DMS) addition decreases H3K9me2 mark enrichment in esophageal squamous cell cancer (ESCC), which inhibits the migration of SHEEC cells.^61^

In conclusion, the knowledge of the relationship between histone methylation and histone succinylation on gene expression and tumourigenesis is still limited. Whether the abnormal expression of the SDH gene is related to histone succinylation and how changes in the SDH gene affect succinylation and diseases should be further researched, as they are meaningful pathways to explore.

#### 3.2.3 Histone lysine desuccinylation

The current research on desuccinylation on histones has mainly found that SIRT5 and SIRT7 in the mammalian sirtuin NAD+‐dependent deacetylase family have desuccinylation activity. Researchers have suggested that SIRT5 is an NAD‐dependent protein lysine desuccinylase and demalonylase in vitro and in vivo.^7^, ^62^ SIRT5 reportedly catalyses the removal of succinylation at H3K9 (H3K9succ) peptide in vitro. Moreover, SIRT5 knockout (KO) mice show increased carbamoyl phosphate synthase 1(CPS1) succinylation marks at Lys1291, which is a known target of SIRT5.^7^ Another study found that SIRT5 is mainly located in the mitochondria and the nucleus. SIRT5 knockout (KO) mouse liver cells and MEFs showed significantly enhanced in mitochondria and cytoplasm and on histones with no significant influence on acetylation, implying that SIRT5 regulates desuccinylation in the nucleus and is independent of acetylation. Moreover, SIRT5 was found by immunofluorescence and confocal microscopy to localize to the nucleus.^13^ SIRT5 certainly desuccinylates at different histone succinyl sites with sequence selectivity in vitro enzymatic assays, such as H2BK116succ and H3K9succ.^63^

SIRT7 was discovered to reside in the nucleolus and is associated with active rRNA genes (rDNA),^64^ histone deacetylation^65^ and histone desuccinylation.^66^ Studies have shown that SIRT7 increases Pol I‐mediated transcription and that SIRT7 KO results in histone hypersuccinylation at specific sites (H3K122 and H4K77) and impairs DNA repair activities.^66^

##### The mechanism of SIRT5‐mediated desuccinylation

Although the current studies of SIRT5 are mainly focused on the activity of enzymes regulating tricarboxylic acid cycle (TCA) and the relationship between metabolism and disease occurrence and development, there are a few studies on the relationship between SIRT5‐mediated histone desuccinylation and disease. However, some studies have explored the mechanism by which SIRT5 binds to proteins for desuccinylation, which may inspire our future research. While studying the structure of SIRT5 to explain its weak deacetylase activity, an arginine residue (Arg105) and tyrosine residue (Tyr102) were discovered in the acyl pocket of SIRT5, which can explain the preference of SIRT5 for negatively charged acyl groups, such as succinyl and malonyl groups.^7^ Through proteomic and statistics analyses, SIRT5 was found to prefer alanine at the +1 site and glycine/alanine at the −1 position^13^ and it was disrupted to recognize histone succinyl substrates when a proline residue was at the +1 site, because it inhibited hydrogen bond formation.^63^ Additionally, SIRT5 recognizes histone succinyl‐lysine residues at the 0, −2, +1 and +3 sites and the main chain hydrogen bonds formed with SIRT5 are highly conserved; these findings were confirmed with in the studies of SIRT5 complex with H3K122succ, H2AK95succ, H2BK120succ and H4K91succ.^63^

These researches reveal the molecular basis of the histone‐desuccinylase activity of SIRT5 to combine specific sequences, which provides insight into SIRT5 desuccinylation activity on histones.

##### SIRT7‐mediated desuccinylation, transcription and DNA repair

Studies have demonstrated that SIRT7 can promote DNA damage repair by catalysing deacetylation in ataxia‐telangiectasia mutated (ATM). Interestingly, an increasing number of studies have further shown that SIRT7 can promote DNA damage repair through histone desuccinylation.

Li et al showed that at the priming stage of the DNA damage response, SIRT7 is recruited to double‐strand break (DSB) sites and catalyses H3K122 desuccinylation depending on poly (adenosine diphosphate (ADP)‐ribose) polymerase 1 (PARP1), which is an essential factor in the priming stage of DNA damage, and both SIRT7 and PARP1 require NAD+ as a coenzyme. H3K122desucc can enhance the binding of histone3 with DNA, thereby strengthening chromatin condensation and DNA damage repair. Moreover, the depletion of SIRT7 in cells reduces desuccinylation, chromatin condensation and DNA damage repair. Because PARPs influence sirtuin activity by restricting NAD+ availability,^67^ PARP1 activation and NAD+ depletion may both inhibit SIRT7 desuccinylation activity, but this supposition remains to be confirmed. However, this study demonstrated that SIRT7 catalyses H3K122desucc, thus playing significant roles in DNA damage repair, antigenotoxicity and cell survival, which provides novel evidence that histone desuccinylation is important in modulating gene expression.^66^ Another study showed that the defective tricarboxylic acid cycle (TCA) metabolism induced by SDH complex subunits with a biallelic deletion genotype perturbs the succinyl‐lysine distribution in chromatin, resulting in histone hypersuccinylation and non‐histone hypersuccinylation, correlating with transcriptional responses.^27^ These results are compatible with previous investigations showing that nucleosome succinylation promotes transcription in vitro. They also demonstrate that defective TCA metabolism reduces DNA repair capacity and genotoxic sensitivity, consistent with identified chromatin hypersuccinylation effects observed in the context of SIRT7 depletion.^66^ Recently, a study demonstrated that a lysine‐to‐glutamate mutation at the H4K77 site, which mimics Ksuc, reduced nucleosome stability and led to defects in DNA damage repair and telomere silencing in vivo.^24^

Accordingly, these findings all show that histone succinylation may modulate genome‐wide transcription and influence DNA repair activities.

## 4 CONCLUSION

The relatively current research on lysine succinylation on histones, a novel protein post‐translational modification, reveals the complexity of succinylation and its function in histones and the nucleosome (Table S1). A few conclusions regarding the regulation and function of histone succinylation can be drawn. There is evidence that histone succinylation influences the structure of the nucleosomes and the expression of genes. Nonetheless, specific genes affected and their relationship with various PTM modifications is not clearly understood. Moreover, histone succinylation is probably mainly regulated by succinyl‐CoA metabolism, and different conditions change the succinylation level. Additionally, the relationship between diseases and histone succinylation has been identified, demonstrating that histone succinylation can be treatment targets in the future. In summary, the studies mentioned in this review reveal glimpses into the complex regulation and function of histone succinylation. Nevertheless, numerous questions await answers through further research.

## CONFLICT OF INTEREST

The authors declare no conflict of interest.

## AUTHOR CONTRIBUTIONS

**Jiayi Liu:** Conceptualization (equal); Data curation (equal); Formal analysis (equal); Investigation (equal); Resources (equal); Writing‐original draft (equal); Writing‐review & editing (equal). **Yu Shangguan:** Resources (equal); Supervision (equal). **Donge Tang:** Funding acquisition (equal); Project administration (equal); Supervision (equal); Validation (equal); Writing‐review & editing (equal). **Yong Dai:** Funding acquisition (equal); Project administration (equal); Writing‐review & editing (equal).

## Supporting information

Table S1

## Data Availability

Data sharing is not applicable to this review as no new data were created in this study.
