# Supplementary material for: Histone succinylation and its function on the nucleosome
Source: J Cell Mol Med. 2021 Jun 23;25(15):7101–9. doi: 10.1111/jcmm.16676 (PMC8335665; doi:10.1111/jcmm.16676)
Supplement: Supplementary file 1 — Table S1 [file JCMM-25-7101-s001.docx]

| Identified Sites | Functions | Regulation | Reference |
| --- | --- | --- | --- |
| H4K77E  H3K79E | reducing the stability of the nucleosomes on chromatin structure  loss of silencing of both telomeres and rDNA | mutants Saccharomyces cerevisiae | ^5, 23, 25^ |
| H4K77suc | interfering with DNA and H2A-H2B dimer assembly | mutant Saccharomyces cerevisiae | ^24, 25^ |
| H2BK37E  H2BK34suc | affecting the interaction of DNA with histones and transcription | Mutant and site-specific succinylation | ^25^ |
| H3K79suc | promoting tumour growing | KAT2A | ^37^ |
|  | promoting YWHAZ and 14-3-3ζ expression and preventing β-catenin degradation | KAT2A | ^55^ |
|  | promoting HBV cccDNA replication and cccDNA microchromosomes epigenetic modification | KAT2A | ^58^ |
| H3K122suc | promoting transcription | P300/CBP, HAT1 | ^28, 49^ |
| H3K122desucc | modulating gene-wide transcription and influencing DNA repair activities | SIRT7 | ^66^ |

Table S1．Function and regulation of identified Ksuc sites^^[[1]](#footnote-1)^^

1. Table S1 shows the identified the function and regulation of histone succinylation (Ksuc) sites. [↑](#footnote-ref-1)
